# Research progress on complications of *B*rucellosis

**DOI:** 10.3389/fcimb.2023.1136674

**Published:** 2023-03-31

**Authors:** Min Jin, Zixu Fan, Ruifang Gao, Xingnan Li, Zhixiang Gao, Zhanli Wang

**Affiliations:** ^1^ School of Public Health, Baotou Medical College, Baotou, China; ^2^ Collaborative Innovation Center of Zoonotic Diseases and Translational Medicine, Baotou Medical College, Baotou, China; ^3^ School of Public Health, Inner Mongolia Medical University, Hohhot, China; ^4^ Inner Mongolia Key Laboratory of Disease-Related Biomarkers, The Second Affiliated Hospital, Baotou Medical College, Baotou, China

**Keywords:** brucellosis, complications, osteoarthritis, endocarditis, meningitis

## Abstract

*B*rucellosis is a common zoonotic disease that is widely spread worldwide and poses a major threat to human health. Clinically, it often presents with non-specific symptoms such as fever, excessive sweating, malaise, myalgia, arthralgia, loss of appetite, weight loss, and enlarged liver, spleen and lymph nodes. The disease has a long and recurrent course, often accumulating in multiple systems and organs. Of these, osteoarticular involvement is the most common complication, with a prevalence of approximately 2-77%, usually manifesting as spondylitis, sacroiliac arthritis and peripheral arthritis. Hepatosplenomegaly is seen in about 50% of patients with *b*rucellosis, and gastrointestinal disturbances such as abdominal pain, nausea, and vomiting are common. Although respiratory involvement is less common, pneumonia, pleurisy, pleural effusion, and pulmonary nodules have been reported. Besides, approximately 2-20% of cases involve infections of the male genitourinary system, mainly manifesting as unilateral epididymal-orchitis and orchitis. The most serious complication facing *b*rucellosis is cardiovascular involvement, and although the overall mortality rate of *b*rucellosis is about 1% and the incidence of *b*rucellosis endocarditis is less than 2%, more than 80% of deaths are associated with endocarditis. Furthermore, *b*rucellosis is complicated by hematologic disease, with anemia occurring in approximately 20-53% of children during the acute phase. In addition, the neurological incidence of *b*rucellosis is about 0.5-25%, mainly manifested as meningitis. In this study, we review the multisystem complications of brucellosis with the aim of improving early diagnosis, timely treatment and prevention of long-term complications.

## 1 Introduction

*B*rucellosis is a common zoonotic disease caused by the Gram-negative, parthenogenic, intracellular parasitic Brucella, with approximately 500,000 new cases each year. The disease was discovered in 1860 by a British physician JA Marston on the Mediterranean island of Maltese, classified as an independent infectious disease, and named “Mediterranean flaccid fever” and “Maltese fever” In 1887, British army doctor David Bruce first isolated “Micrococcus Maltese” from the spleen of a soldier who died on Malta island, identifying the disease’s causative agent. Subsequently, the disease *b*rucellosis was named in honor of Bruce (Shakir, 2021). In 1985, the World Health Organization (WHO) Expert Committee on *B*rucellosis classified the genus *B*rucella into six biological species and 19 biological subtypes (classical biotyping): *B*. melitensis, *B*. abortus, *B*. suis, *B*. ovis, *B*. neotomae, and *B*. canis (Jiao et al., 2021). The most common pathogenic species for human brucellosis is *B*. melitensis, while *B*. abortus is the weakest type (Deng et al., 2019). *B*rucella is an expanding genus. With advances in microbiology, genomics, molecular biology, and other technologies, several natural hosts have been identified, such as dolphins, whales, camels, bison, foxes, baboons, bats, and frogs. Interestingly, the emergence of new species has weakened the value of classical biotypes. *B*rucella can enter the body through direct contact, conjunctival inoculation, gastrointestinal tract, respiratory tract, and biological transmission (Whatmore and Foster, 2021).

*B*rucella is a group of micro-globular or short rod-shaped bacteria (0.5–0.7/0.6–1.5 μm), nonmotile, and slow-growing (Deng et al., 2019). Without classical virulence factors, such as flagella, spores, plasmid, and exotoxin.,Lipopolysaccharide (LPS), outer membrane protein (OMP), Type IV secretion system (T4SS), and BvrR/BvrS system contribute to virulence of Brucella. The pathogenicity depends on its ability to multiply and survive within macrophages (Głowacka et al., 2018). The bacteria has strong resistance in the natural environment and is sensitive to light, heat, acid, and common disinfectants. It can survive for about 120 days in the secretions of sick animals, excretion and organs of dead animals, and for 60 days in dairy products (Unuvar et al., 2019). *B*rucella can enter the body through direct contact, conjunctival inoculation, gastrointestinal tract, respiratory tract, and biological transmission. While, nosocomial contagion is an important mode of infection. In particular, laboratory workers are likely to acquire the bacteria through aerosols or direct contact (Li et al., 2020).The disease is common in the Middle East, Asia, Africa, South and Central America, the Mediterranean, and the Caribbean. More than 170 countries and territories worldwide have reported cases of brucellosis, with outbreaks in Syria, Mexico, Peru, Argentina and Africa were severely affected (Pappas et al., 2006; Giambartolomei and Delpino, 2019). Clinically, *b*rucellosis is manifested with non-specific symptoms, such as fever, excessive sweating, malaise, loss of appetite, testicular enlargement, joint pain, skin rash, and enlarged liver, spleen, and lymph nodes. Although complicated symptoms, fever and arthralgia are the main manifestations (Dean et al., 2012).

The diagnosis of *b*rucellosis depends mainly on the epidemiological history of the patient, clinical manifestations, serological tests and bacterial cultures. The rose Bengal test (RBT) is commonly used for primary screening because of its high sensitivity, low cost and simplicity. The standard agglutination test (SAT) titer ≥1:160 and the 2-mercaptoethanol test (2-ME) titer ≥1:80 are considered diagnostic when coupled with a compatible clinical presentation. Of course, the results of both SAT and 2-ME experiments are related to the characteristics of the patient population and the quality of the reagents used. A SAT ≥1:320 is still recommended as a cut-off value for serological diagnosis of human brucellosis in endemic areas. In areas with low disease prevalence, the titre range of 1:40 to 1:80 in 2-ME experiment can be considered for the diagnosis of *b*rucellosis *(* Yagupsky et al., 2019).

*B*rucellosis is a significant public health issue in developing countries, posing a major threat to human health the entire social and economic development. Because its clinical symptoms are not specific, it is easy to be misdiagnosed and that leads to chronic phase. What is more, If treatment is not taken in the acute phase, various complications will occur, resulting in disability, skeletal deformity, and even death. Besides, *b*rucellosis may accumulate complications of the locomotor-osteoarticular system, digestive system, respiratory system, genitourinary system, cardiovascular system, nervous system, and blood, posing a major threat to human health the entire social and economic development. This article provides a systematic review of *b*rucellosis-related complications.

## 2 Motion-osteoarticular system

Involvement of bone and joint is the most common complication of *b*rucellosis, with a prevalence of approximately 2-77%. *B*rucella osteoarthropathy usually presents as spondylitis, sacroiliac arthritis, peripheral arthritis and osteomyelitis, while bursitis and tenosynovitis are rare. 2-60% prevalence of *B*rucella spondylitis, 2-45% prevalence of sacroiliac arthritis, and 14-26% prevalence of peripheral arthritis were reported (Unuvar et al., 2019).

*B*rucella spondylitis is the most common and severe manifestation of the osteoarthritis system, prevalent in elderly patients, often presenting as chronic back pain with fever, systemic symptoms, and dysfunction and teratogenicity in advanced stages of the disease. Spondylitis is dominated by involvement of the lumbar spine, followed by the thoracic and cervical spine (60% in the lumbar spine, 19% in the thoracic spine, and 12% in the cervical spine). The lumbar spine, an important weight-bearing structure of the human spine, is highly mobile and contains many cancellous bones and a rich venous plexus where bacterial emboli are more likely to lodge. The lumbar spine mainly presents with focal lesions, with the L4 and L5 vertebrae predominantly involved, and discontinuous multi-site vertebral segmental injuries are less common (Unuvar et al., 2019). Spondylodiscitis is common at the L3-L4, L4-L5 and L5-S1 levels and can also accumulate in multiple regions of the cervical, thoracic and lumbar spine (Ma et al., 2021). Complex *b*rucellosis spondylitis is severe and the infection can spread from the vertebrae to adjacent tissues such as the epidural, prevertebral and paravertebral tissues, major lumbar muscles and nerve roots. These manifestations can lead to spinal stenosis and trigger spinal cord and nerve compression with muscle spasm, motor weakness, sensory abnormalities and, in severe cases, neurological complications, such as paraplegia (Ulu-Kilic et al., 2014). High-resolution magnetic resonance imaging (MRI) is more useful in diagnosing brucellosis spondylitis (Charalambides et al., 2010).

Sacroiliac arthritis is more prevalent in patients infected with sheep seed bacteria, and its presentation can be unilateral or bilateral, predominantly in young people with significant pain. The incidence was higher in the 15-35 years age group, accounting for 55.1% (Hizel et al., 2007). It is usually accompanied by fever, low back pain and hip pain. Examination revealed positive straight-leg-rise maneuvers test and FABER test, and may be lead to easily misdiagnosed easily. Bone scans, MRIs, and arthrocentesis can avoid misdiagnosis (Dayan et al., 2009). Several studies have confirmed that elevated C-reactive protein is positively associated with the presence of sacroiliitis, C-reactive protein is expected to be a new diagnostic marker (Hizel et al., 2007).

*B*rucellosis complicated with peripheral arthritis is more common in children and adolescents, often accumulated knee joint, hip joint, ankle joint, etc. The typical symptoms of knee arthritis are swelling and pain in one joint (Zamani et al., 2011). The initial symptoms of hip arthritis are not obvious, but as the disease progresses, the pain worsens, accompanied by limited movement and deformity of the lower limbs. Physical examination showed deep tenderness and percussive pain at the lesion site, accompanied by a positive Tomas sign and positive “4” test; diagnosis and treatment are challenging. Delayed treatment can lead to severe complications, such as femoral head dislocation and ischemic necrosis (Jahmani et al., 2021). Shoulder arthritis is common in the elderly, with symptoms lasting long and often requiring an extended period to recover from joint damage. On the other hand, wrist, and ankle arthritis is relatively mild, and the prognosis is satisfactory (Bosilkovski et al., 2016).

Osteomyelitis is usually hematogenous. When *B*rucella invades the body, it can enter the bone marrow through blood-borne transmission and invade the bone marrow and bone cortex in a big way. Patients may present with recurrent high fever (wave fever), malaise, muscle aches, and joint pain. In adults, osteomyelitis accumulates in the spine and is characterized by spinal fever and pain, while in adolescents, osteomyelitis of the knee (Mete et al., 2012).

## 3 Digestive system

Gastrointestinal symptoms are common in patients with *b*rucellosis and can manifest as decreased appetite, nausea, vomiting, abdominal pain, diarrhea and constipation, and hepatosplenomegaly in about 50% of patients with *b*rucellosis. If gastrointestinal disturbances are present, gastrointestinal complications should be considered (Dean et al., 2012).

The liver is the most extensive reticuloendothelial phagocytic system in the body and is capable of a rapid and controlled response to invasion by pathogenic microorganisms. *B*rucella, however, can use the immune tolerance of the liver to evade the immune response and persist in the host. *B*rucella can stimulate hepatic shape cell activation and secretion of collagen, forming scar tissue and leading to chronic fibrosis or cirrhosis. Liver function is usually normal in *B*rucella-infected individuals, with the most common abnormalities manifesting as increases in transaminases and alkaline phosphatase that are not specific. However, all cases of elevated liver enzymes cannot be classified as liver involvement, and acute hepatitis due to *b*rucellosis is not too common (Giambartolomei and Delpino, 2019).

Reductions in hemoglobin and platelets often accompany the digestive complications caused by brucellosis. Patients mostly consume raw milk, and on examination they may present with fever, excessive sweating, nausea, vomiting, abdominal pain, and other symptoms such as right quadrant tenderness, hepatosplenomegaly, and yellowing of the sclera. Serological tests and imaging can confirm the diagnosis, and abdominal computed tomography (CT) is more sensitive than abdominal ultrasound (Denk and Ozden, 2015). In a study of 251 patients with brucellosis, Pourbagher et al. identified 21 (8.4%) patients with splenomegaly, 15 (6%) patients with hepatomegaly, 4 (1.6%) patients with splenic abscess, 2 (0.8%) patients with splenic cyst, 2 (0.8%) patients with acute appendicitis, 1 patient (0.4%) with acute acalculous cholecystitis (Pourbagher et al., 2006).

In areas where *b*rucellosis is endemic, patients with cirrhosis may be complicated by bacterial peritonitis. However, Brucella infection can also directly trigger ascites, which is extremely rare. Infection is usually detected by lymphocytic exudate, which may be related to the immune response of the abdominal mononuclear phagocytic system (Kantarçeken et al., 2005). In brucellosis combined with liver abscess, patients may present with intermittent fever, nausea, vomiting, right upper abdominal pain, and right upper abdominal peritoneal irritation on examination (Le Moigne et al., 2016). Patients with concurrent splenic abscess often have fever, arthralgia, and persistent dull pain in the left upper abdomen. The development of splenic abscesses may be associated with *B*rucella endocarditis. In hepatosplenic abscesses, routine antimicrobial therapy and puncture drainage are adequate measures (Deveer et al., 2013).

*B*rucellosis with acute cholecystitis is usually non-chronic and the pathogenesis is unknown. Usually, patients present on examination with fever, malaise, right upper abdominal pain, sometimes constipation and marked abdominal pressure with rebound pain, and Murphy’s sign is usually positive (Hariz et al., 2019). *B*rucella may also enter the pancreas through the biliary system and bloodstream, leading to acute pancreatitis, usually with fever, abdominal pain, nausea, vomiting, extensive mucosal yellowing of the skin and pancreatic edema, and elevated hepatobiliary enzymes may be associated with acute pancreatitis (Berber et al., 2014).

## 4 Respiratory system

Respiratory infections of *b*rucellosis are rare and most are reported as clinical cases; pneumonia, pleurisy, pulmonary nodules, pulmonary granuloma, pleural effusion, lung abscess, thoracic abscess, pneumothorax, lymph node enlargement and mediastinal disease have been reported (Pappas et al., 2003; Olukman, 2008).

In a retrospective study, Georgios Pappas et al. found respiratory involvement in only 37 of 450 patients with *b*rucellosis. Patients presented mainly with fever and cough, with individual symptoms such as dyspnea, manifesting as lobar pneumonia, bronchopneumonia, pleural effusion and hilar lymph node enlargement (Pappas et al., 2003). Pulmonary *b*rucellosis may be associated with inhalation of contaminated aerosols and transmission of bacteraemia. However, it is rarely severe and effective with conventional therapy for simple brucellosis (Olukman, 2008). Erdem et al. retrospectively studied respiratory infections in patients with *b*rucellosis in Turkey over a ten-year period from 2002 to 2012. Out of 133 patients with pulmonary brucellosis, 123 (92.5%) presented with acute infection and most presented with pneumonia (Erdem et al., 2014). Most of the patients with pneumonia had a history of consuming raw, unpasteurized milk or dairy products. The clinical presentation was mainly fever and cough, with an audible wet rales in the lungs, which could be further confirmed by imaging. Serologic testing or blood cultures confirm *B*rucella infection, and laboratory tests may be accompanied by elevated C-reactive protein, erythrocyte sedimentation rate, calcitoninogen, and other inflammatory markers (Xie et al., 2019). In addition, massive infiltration of inflammatory cells in the lungs may lead to massive pulmonary changes and pleural effusions (Singh et al., 2005).

In pulmonary nodules complicated by *b*rucellosis, patients may present with bilateral pulmonary nodules on chest radiographs and CT, in addition to chest pain (Sevilla López et al., 2011). *B*rucellosis can also be complicated by pleural effusion, and in addition to the typical symptoms of pleurisy, such as chest pain, patients may present with cough and dyspnea. Pleural effusions are mainly lymphatic exudate, high in protein, low in pH and sugar, and may be positive for *B*rucella culture. Treatment may include antimicrobial and thoracentesis tube drainage and, if necessary, pulmonary cortical debridement (Erdem et al., 2014). *B*rucellosis is easily overlooked when it accumulates in the lungs, and clinical workers should pay attention to the patient’s epidemiologic history, serologic testing, and imaging for comprehensive evaluation, as well as for differentiation from tuberculosis and other respiratory diseases, with bronchoscopy feasible when necessary (Pericherla et al., 2021).

## 5 Genitourinary system

Approximately 2-20% of *b*rucellosis cases involve the genitourinary system and often present clinically with fever, swelling of the epididymis, and scrotal pain. The most common complications of genitourinary *b*rucellosis are unilateral epididymal-orchitis and orchitis, followed by prostatitis, cystitis, vaginitis and tubo-ovarian abscesses, with less frequent renal involvement (Batirel et al., 2020).

*B*rucellosis with testicular infection is predominant in the acute phase. In a retrospective study, Zhou Yan et al. found complications of genitourinary injury in 22 of 801 patients admitted with brucellosis over a 10-year period. Male patients were mostly complicated by orchitis, epididymal-orchitis, prostatitis and urethral stricture, mainly manifested by fever, testicular swelling, pain and difficulty in urination. Only one woman was complicated by vaginitis and cervicitis, manifested by increased leucorrhea, menstrual irregularities and lower abdominal distension (Zhou et al., 2020). Epididymitis often coexists with orchitis, called epididymal-orchitis, and is clinically characterized by persistent fever, acute scrotal pain, swelling and congestion, and may be accompanied by rare testicular abscesses, atrophy and oligospermia (Gozdas and Bal, 2020).Safwat et al.found a significantly impaired reproductive system and a higher prevalence of erectile dysfunction (ED) in patients with chronic *b*rucellosis, accounting for 70% of cases, and associated with testicular atrophy and serum decreased serum testosterone levels (Safwat et al., 2018).

The accumulated kidneys of brucellosis can be divided into three types. First, acute interstitial nephritis or pyelonephritis occurring in the acute phase of *b*rucellosis, mostly associated with hematuria, proteinuria or pusuria. Second chronic *b*rucellosis with renal involvement, which can lead to chronic granulomatous interstitial nephritis with caseous necrosis and calcification, similar to renal tuberculosis or chronic nonspecific pyelonephritis. Third, *b*rucellosis endocarditis with renal involvement is associated (Conkar et al., 2018). Patients with renal involvement have varying degrees of hematuria and proteinuria in addition to the typical symptoms of *b*rucellosis, such as fever, hyperhidrosis, malaise, and loss of appetite (Ceylan et al., 2009). If the lesions accumulate in the glomerulus, patients tend to present with abnormal urinary sediment, proteinuria or azotemia. The pathogenesis of this disease is usually related to the deposition of circulating immune complexes (Kusztal et al., 2007). Acute renal failure due to brucellosis is rare and is mainly caused by acute interstitial nephritis caused by direct invasion of the kidney by *B*rucella infection. Patients may present with oliguria or anuria and significant elevation of urea nitrogen and creatinine (Ghanei et al., 2009).

## 6 Cardiovascular system

Patients with brucellosis are usually associated with varying degrees of ventricular diastolic dysfunction and myocardial injury. Recurrent chronic infection with *B*rucella can lead to vascular endothelial dysfunction and promote atherosclerosis, leading to various cardiovascular complications (Togan et al., 2015).

Infective endocarditis is the most common complication of *b*rucellosis combined with the cardiovascular system, although its incidence is less than <2% of patients with brucellosis (Ece et al., 2020). Endocarditis often invades the aortic and mitral valves, and patients often present with fever, malaise, and chest pain, along with elevated C-reactive protein or erythrocyte sedimentation rate. Infective endocarditis can lead to venous thrombosis, organ embolism, and heart failure, which can be fatal. Although the overall mortality rate of brucellosis is approximately 1%, >80% of *b*rucellosis deaths are associated with endocarditis (Tuncer et al., 2008; Açar et al., 2015). Studies have shown that embolic events are present in 22%-43% of patients with infective endocarditis. Acute myocardial infarction and ischemic stroke due to endocarditis caused by *B*rucella infection are usually associated with septic artery embolism. The most common site of infected coronary embolism is the left anterior descending branch; however, coronary angiography performed during active endocarditis may lead to systemic embolism (Açar et al., 2015; Randa et al., 2021). *B*rucella infection can also lead to aortic involvement, and extensive diagnostic testing for *B*rucella aortic involvement and aneurysm formation should be performed in patients over 50 years of age with positive Brucella blood cultures and presenting with fever, back pain, or chest pain (Cascio et al., 2012).

*B*rucella infection can also lead to arrhythmias or coronary ischemic changes. Lu et al. found abnormalities in 31/108 (28.7%) patients with brucellosis. The ECG showed sinus bradycardia, precontraction of the ventricles, conduction block, ST-T abnormalities, Q-wave abnormalities and poor R-wave progression (Lu et al., 2021).

## 7 Blood system

Hematologic abnormalities in *b*rucellosis may be associated with altered iron metabolism due to infection, hypersplenism, phagocytosis, myelosuppression, diffuse anticoagulation, and autoimmune hemolysis (Okur et al., 2012).

*B*rucellosis is complicated by hematologic disease and most commonly occurs in pediatric patients in the acute phase; patients may have hepatosplenomegaly and lymph node enlargement. Anemia is the most common hematologic complication, occurring in approximately 20-53% of children; the incidence of leukopenia is approximately 8-38%; thrombocytopenia 2-16%; and the incidence of pancytopenia is usually less than 10%.Justman et al. found that in 511 brucellosis disease, 68 (13%) were anemic, 144 (28%) were leukopenic, 74 (14%) were thrombocytopenic, and 9 (2%) were allocytopenic (Justman et al., 2018).

Patients with *b*rucellosis complicated with pancytopenia often present with fever, loss of appetite, joint pain and other symptoms, and bone marrow aspiration can show histiocytic hemophagocytosis and granuloma formation (Sari et al., 2008). *B*rucella infection can also be complicated by secondary thrombocytopenic purpura, which is mainly manifested as fever and ecchymosis in the extremities, and may be accompanied by severe thrombocytopenia in laboratory tests (Bhasin et al., 2021). *B*rucellosis can also cause hemophagocytic lymphohistiocytosis (HLH), in which patients may present with fever, hepatosplenomegaly, cytopenia, hypofibrinogenemia, hypertriglyceridemia, and hyposideremia. Histiocytic proliferation and phagocytosis were observed in bone marrow aspirates (Mittal et al., 2021). Meanwhile, Bakri et al. reported the first case of bone marrow fibrosis caused by *b*rucellosis leading to pancytopenia (Bakri et al., 2010). *B*rucellosis may also be associated with hematological malignancies. In addition, Brucella infection can also lead to myelodysplastic syndrome (MDS) and gammopathy (Wang et al., 2020).

## 8 Nervous system

The incidence of brucellosis in the nervous system is about 0.5-25%, and its pathogenesis may be related to the direct invasion of intracellular microorganisms or the immune mechanism of neuropathological changes caused by infection (Kanjo et al., 2021).

Neurobrucellosis (NB) presents in three forms: meningitis, chronic peripheral disease and diffuse central nervous system disease (Gul et al., 2009). The diagnosis depends on the patient’s clinical presentation, cerebrospinal fluid testing, imaging, serological testing, and bacterial culture. Cerebrospinal fluid testing revealed lymphocytosis, an increased protein level, and a decreased glucose concentration. In patients with abnormal signs and suspected *b*rucellosis, cranial MRI is preferred (Díaz-Vintimilla et al., 2021). Meningitis is the most common manifestation of neurobrucellosis. In addition, meningoencephalitis, myelitis, radiculitis, cranial nerve involvement, cerebrovascular disease, brain abscess, intracranial hypertension, hydrocephalus, epidural abscess, demyelinating disease, Guillain-Barre syndrome (GBS), cerebral venous thrombosis, paraplegia, and aphasia have been reported (Maji et al., 2020).

Patients with neurobrucellosis may present with fever, headache, accompanied by meningeal irritation, hyperalgesia and confusion [73]. Infection of the central nervous system in *b*rucellosis can also lead to the syndrome of inappropriate secretion of antidiuretic hormone (SIADH), which is also prevalent in *b*rucellosis. Bala et al. detected SIADH in 35 of 160 (21.9%) children and adolescents with *b*rucellosis. The pathogenesis of SIADH remains unclear and may be related to hypoxia and hypovolemia (Bala et al., 2016).

## 9 Other

The prevalence of *b*rucellosis complicated with skin lesions is about 5-17%. It is broadly divided into four categories: disseminated papules, nodular rashes (most common), erythema nodosum lesions, generalized maculopapules, and generalized purpura. The pathogenesis may be related to hypersensitivity, immune complex deposition, vaccination or blood transmission of *B*rucella (Al Jasser and Al Ajroush, 2012; Uçmak et al., 2014).

Rolando et al. found that of 1551 patients with *b*rucellosis admitted over a 26-year period, 52 had ocular *b*rucellosis (3.4%). Among them, 43 cases (82.7%) had uveitis. Ocular involvement is common in the chronic phase of *b*rucellosis, especially in young women aged 16-35 years, who often present with blurred vision (Rolando et al., 2008). *B*rucella infection can also cause cervical *b*rucellosis lymphadenopathy, and patients usually complain of neck swelling. Submandibular mass and local lymph node necrosis were seen on neck CT, which required needle biopsy to confirm infection to rule out malignancy (Yilmaz et al., 2009). In infectious thyroiditis caused by *b*rucellosis, patients often have anterior neck pain and swelling, accompanied by sore throat, mild dysphagia and hoarseness, and goiter can be seen on physical examination. If left untreated, a thyroid abscess may develop. Thyroid ultrasound and thyroid function measurement can complement the diagnosis (Cvetkova et al., 2019).

Breast involvement after *B*rucella infection is less common. Nonetheless, it can be complicated by mastitis and breast abscesses, and patients may present with fever, breast pain, swelling, and patchy erythema around the areola. Breast ultrasound, pus culture, and needle biopsy can help diagnose infection. Although breast infection is rare in lactating women, pregnancy and a history of breast augmentation may be predisposing factors (Jensenius et al., 2008).

## 10 Conclusion

*B*rucellosis is a systemic disease that may accumulate in multiple tissues and organs of the body, consisting of the musculoskeletal system, reproductive system, central nervous system, liver, heart, and lungs (see **Table 1** for details of involvement/complication rates). The existence of these complications brings great challenges to diagnosis and treatment. A diagnosis should be made with a comprehensive judgment on multiple examinations and test results, so as to improve the diagnostic accuracy and avoid misdiagnosis or underdiagnosis. On the other hand, a reasonable therapy plan should be formulated according to the state of illness and the involved tissues and organs. It is recommended to take two or more antibiotics combined treatment, reducing delayed treatment and misdiagnosis, preventing the occurrence of chronicity, improving the treatment effect of *b*rucellosis, and avoiding recurrence and complications.

**Table 1:** Multisystem complications of *b*rucellosis and their involvement/complication rates.

System	Complications	complication rate	Original Citation
Motion-osteoarticular system	Spondylitis	2-60%	(Unuvar et al., 2019)
	Sacroiliac arthritis	2-45%	(Unuvar et al., 2019)
	Peripheral arthritis	14-26%	(Unuvar et al., 2019)
	Intervertebral discitis	NA	(Charalambides et al., 2010)
Digestive System	Enlarged liver and spleen	50%	(Dean et al., 2012)
	Liver abscess	NA	(Le Moigne et al., 2016)
	Splenic abscess	NA	(Deveer et al., 2013)
	Acute cholecystitis	NA	(Hariz et al., 2019)
	Acute pancreatitis	NA	(Berber et al., 2014)
Respiratory System	Pneumonia	NA	(Erdem et al., 2014)
Genitourinary system	Epididymo-orchitis	2-20%	(Batirel et al., 2020)
Cardiovascular System	Endocarditis	2%	(Ece et al., 2020)
	Arrhythmia or coronary ischemia	28.7%	(Lu et al., 2021)
blood system	Anemia	20-53%	(Justman et al., 2018)
	Leukopenia	8-38%	(Justman et al., 2018)
	Thrombocytopenia	2–16%	(Justman et al., 2018)
	Complete blood cytopenia	10%	(Justman et al., 2018)
	HLH	NA	(Mittal et al., 2021)
Nervous System	Meningitis	0.5–25%	(Gul et al., 2009; Díaz-Vintimilla et al., 2021; Kanjo et al., 2021)
Other	skin lesion	5-17%	(Al Jasser and Al Ajroush, 2012; Uçmak et al., 2014)
	Ocular brucellosis	3.4%	(Rolando et al., 2008)
	Thyroiditis	NA	(Cvetkova et al., 2019)

NA means not applicable.

## Author contributions

ZG, ZW, and MJ contributed to the conception and design of this study. ZF, RG, and XL performed the literature collection. MJ and ZF wrote the first draft of the manuscript. All the authors contributed to manuscript revision, read, and approved the version as submitted.
